# Supplementary material for: Genomic characterization of DICER1-associated neoplasms uncovers molecular classes
Source: Nat Commun. 2023 Mar 25;14:1677. doi: 10.1038/s41467-023-37092-w (PMC10039902; doi:10.1038/s41467-023-37092-w)
Supplement: Supplementary file 1 — Supplementary Information [file 41467_2023_37092_MOESM1_ESM.pdf]

Supplementary Information

Kommoss et al., Genomic characterization of DICER1-associated neoplasms uncovers molecular classes

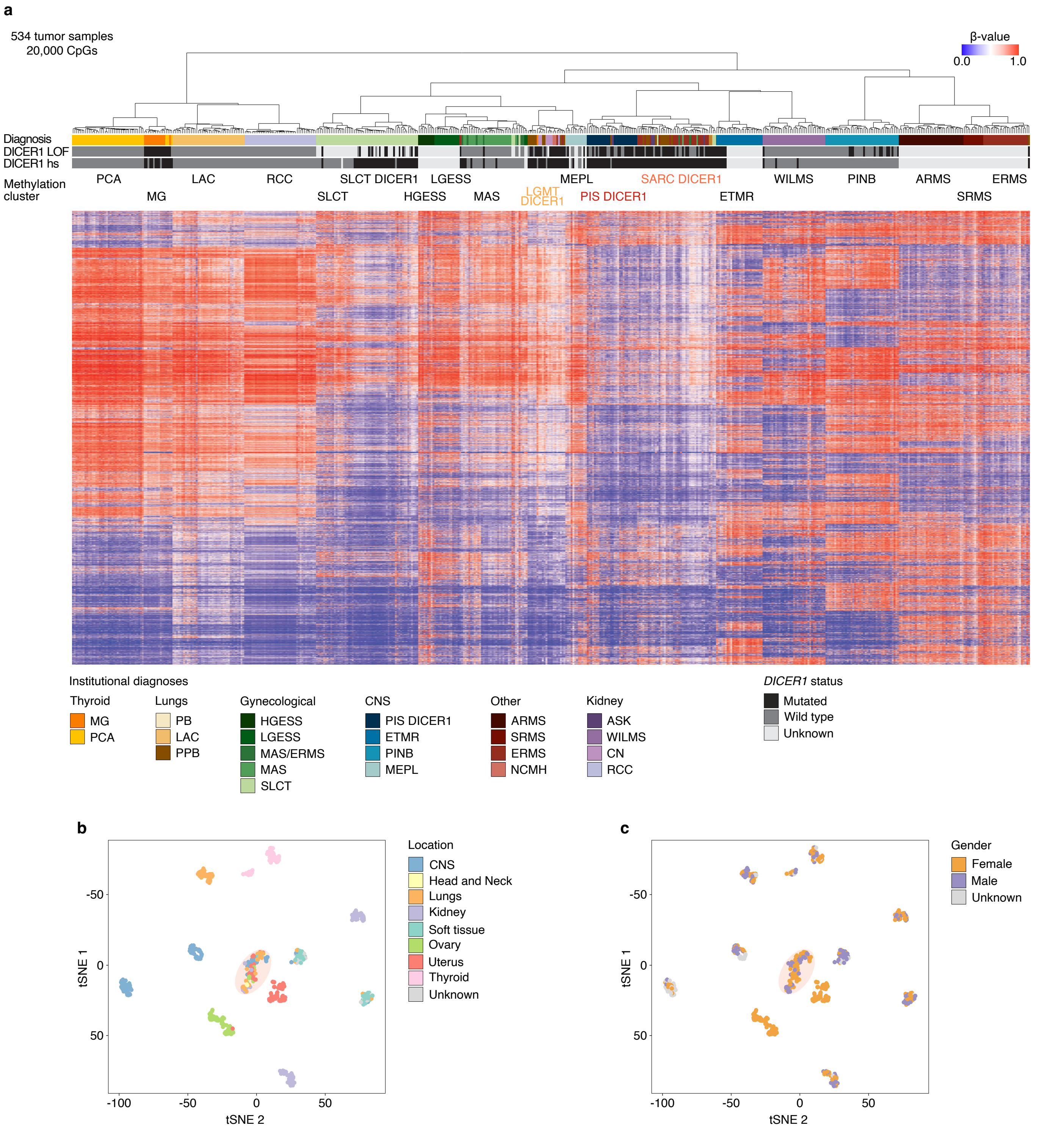

Supplementary Figure 1: a - c, Molecular classification of DICER1-associated neoplasms by DNA methylation analysis (related to Fig. 1). **a**, Unsupervised hierarchical clustering (Euclidean ward) of the 20,000 most differentially methylated CpGs. **b** and **c**, t-SNE dimensionality reduction of the 8,000 most differentially methylated CpGs. The same 534 tumor samples as in Fig. 1a are used. (**a**) Samples are colored according to their institutional diagnoses. Black indicates known DICER1-association, dark-grey indicates no DICER1 association and light-grey resembles unknown DICER1-association. (**b**) Cases are colored according to their anatomical location. (**c**) Cases are colored according to gender.

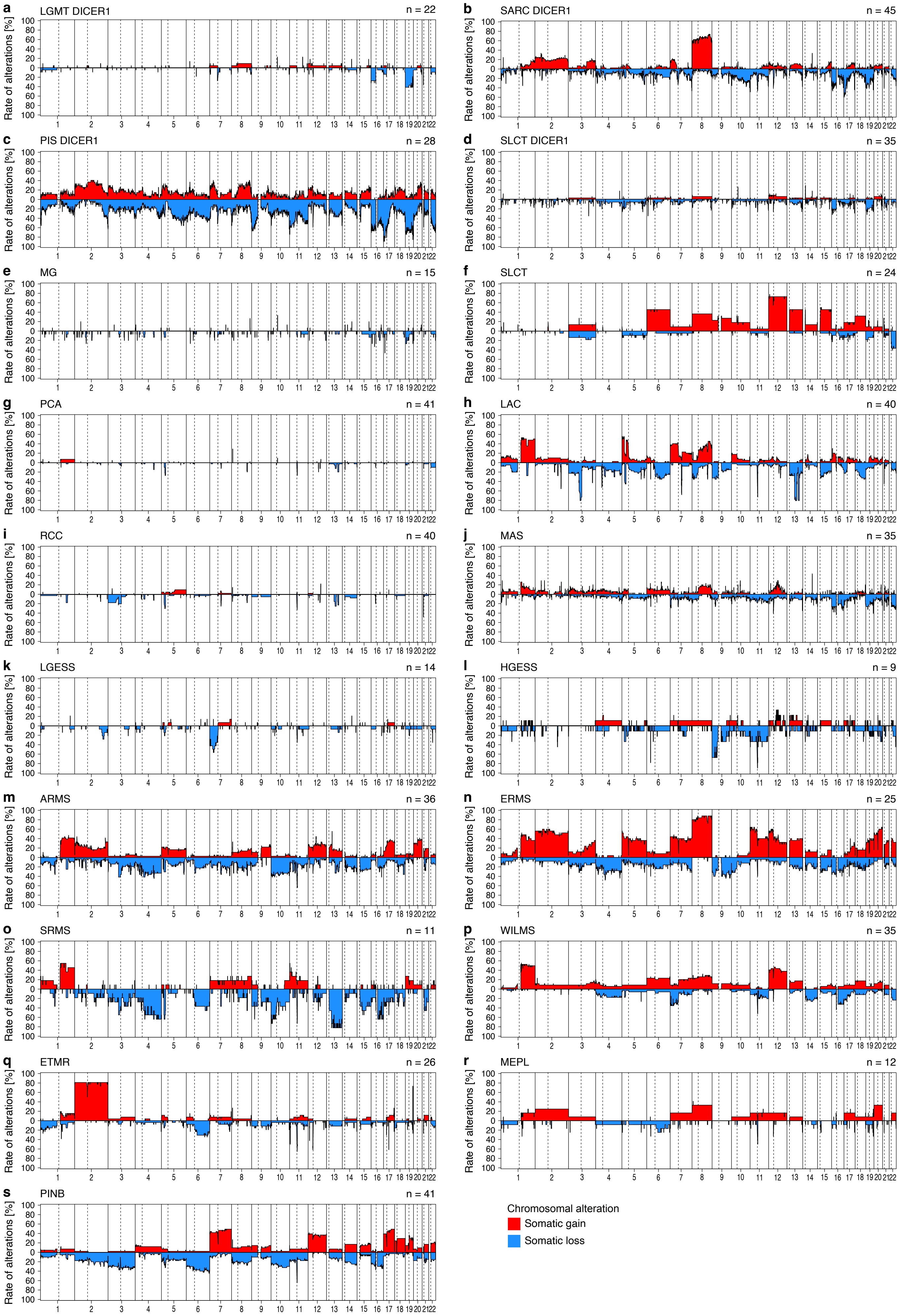

Supplementary Figure 2: Cumulative copy number profiles of molecular classes identified by DNA methylation analysis (Fig. 1a, Supplementary Data 1). a - s, Plots showing the frequency of any chromosomal aberration at the respective loci. Chromosomes are numbered accordingly. Chromosomal gains are depicted in red and losses shown in blue.

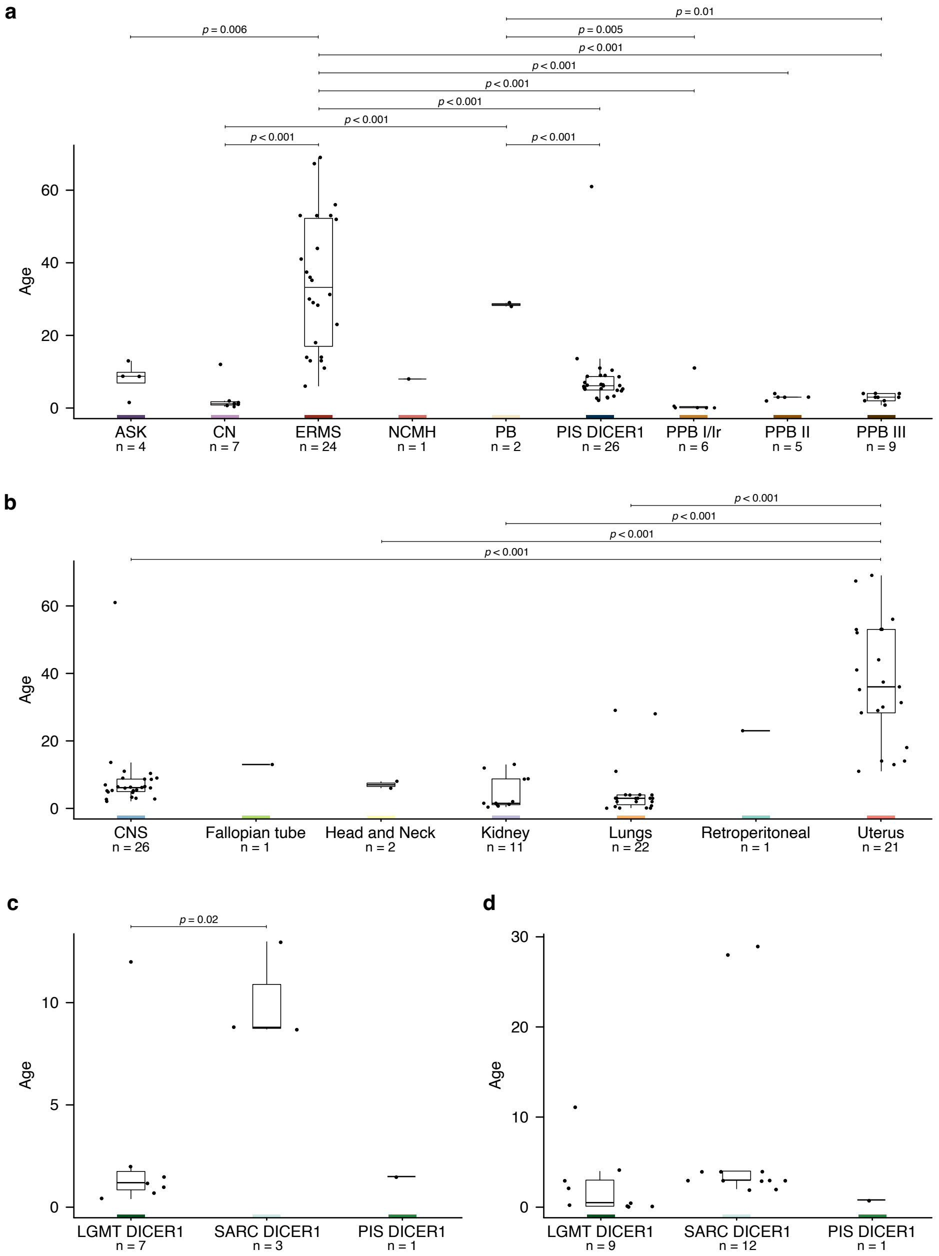

Supplementary Figure 3: Age distribution of mesenchymal tumors with DICER1 alteration. a, Age distribution of patients with LGMT DICER1, SARC DICER1 and PIS DICER1 according to institutional diagnoses. **b**, Age distribution of patients with LGMT DICER1, SARC DICER1 and PIS DICER1 according to tumor location. **c,** Age distribution of patients with kidney tumors according to LGMT DICER1, SARC DICER1 and PIS DICER1 methylation class assignment. **d,** Age distribution of patients with lung tumors according to LGMT DICER1, SARC DICER1 and PIS DICER1 methylation class assignment. Black lines mark the median, edges of boxes denote interquartile range (IGR), and vertical lines indicate 1.5 x IQR. Statistical significance was determined by the Games-Howell post-hoc test.

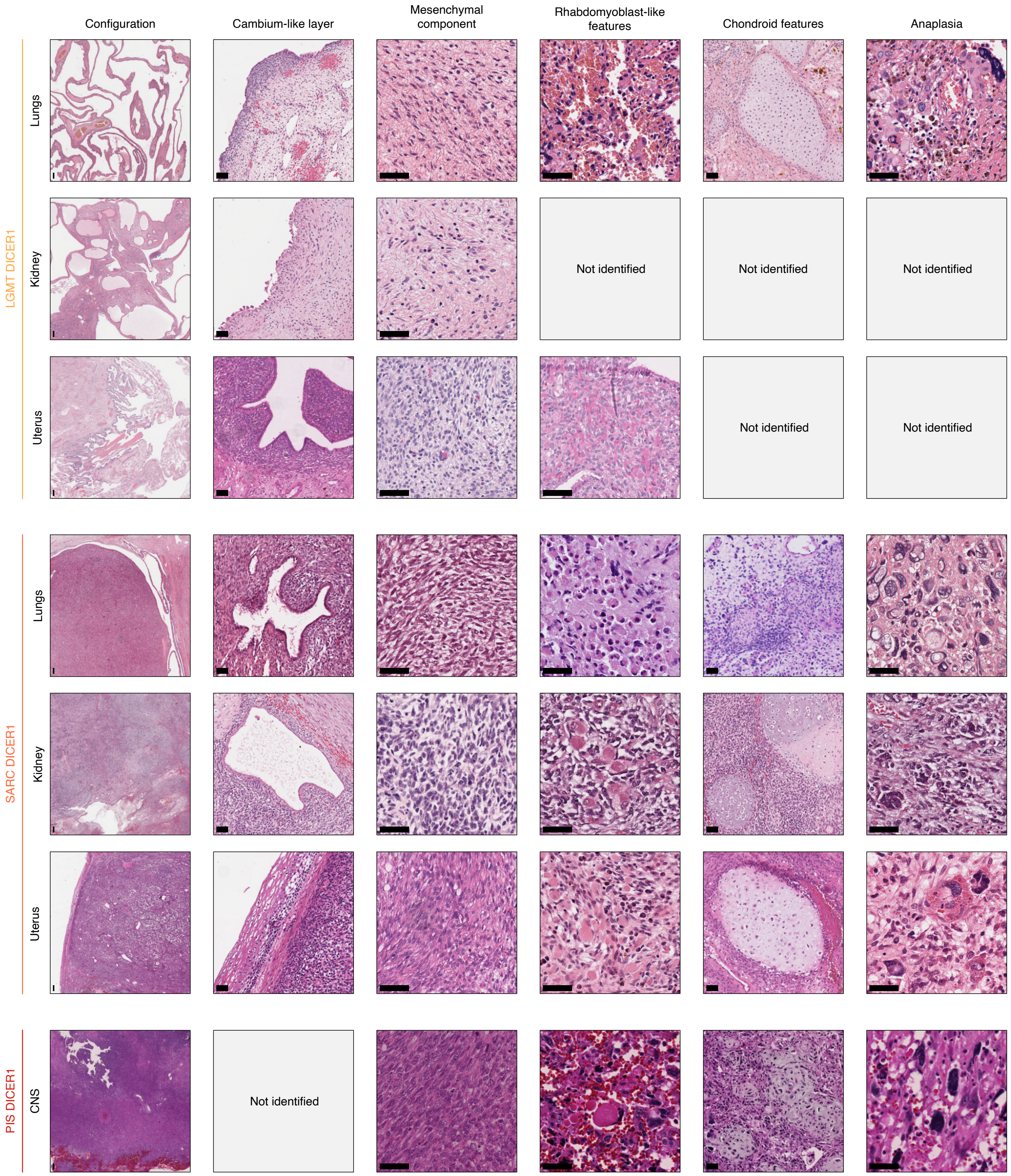

Supplementary Figure 4: Histological features of mesenchymal tumors with DICER1 alteration. Histological features shared across mesenchymal tumors with DICER1 alteration according to methylation class assignment of LGMT DICER1, SARC DICER1 and PIS DICER1 and anatomical location. Hematoxylin & Eosin, bar equals 50 μm.

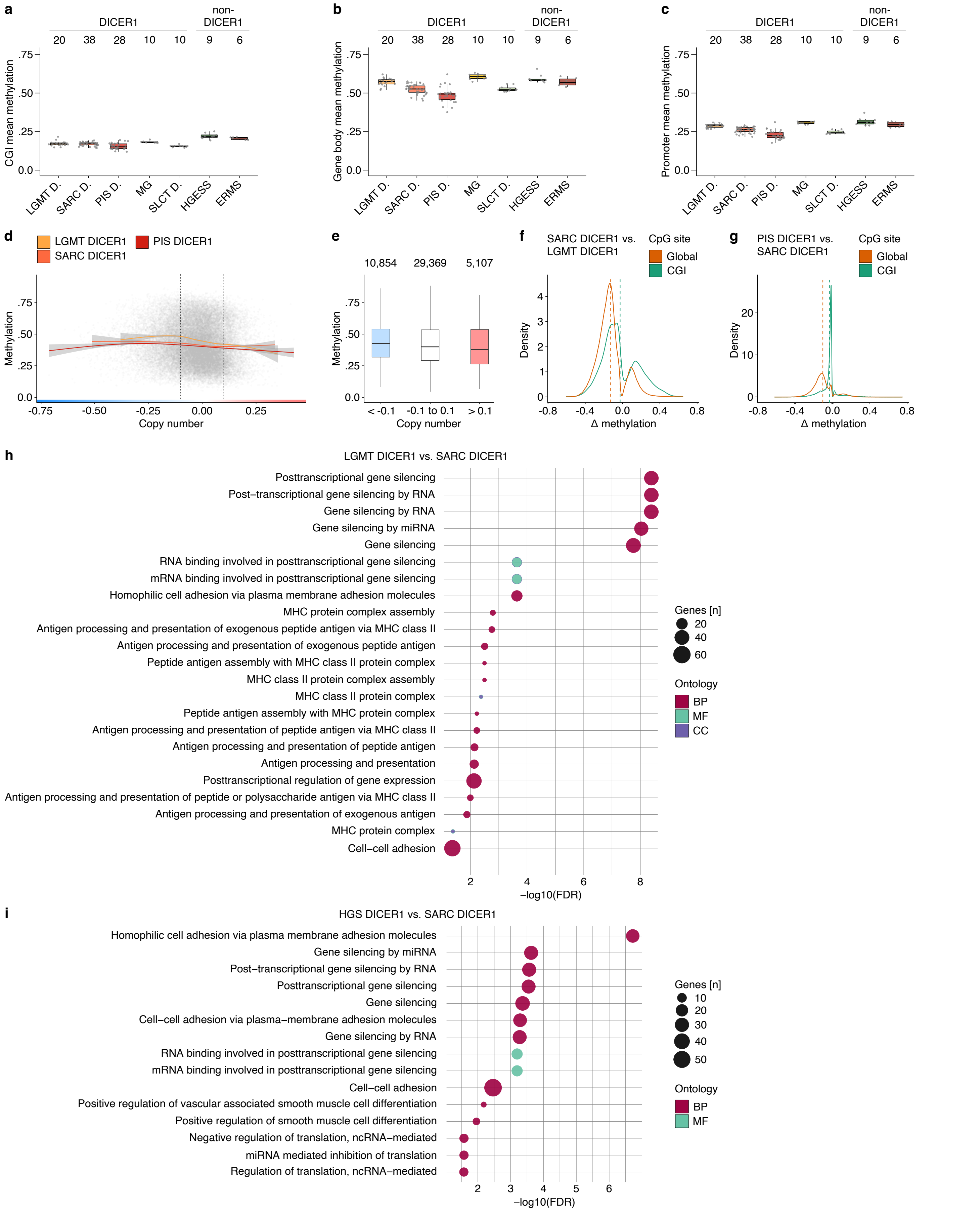

Supplementary Figure 5: Extended DNA methylation analyses of mesenchymal neoplasms with DICER1 alteration. a - c, (**a**) DNA methylation levels of CpG-dense regions in CGIs, (**b**) DNA methylation levels of CpGs in gene bodies and (**c**) DNA methylation levels of CpGs in promoter regions for samples of LGMT DICER1, SARC DICER1 and PIS DICER1, MG and SLCT DICER1, as well as ERMS and HGESS. Black lines mark the median, edges of boxes denote interquartile range (IQR), and vertical lines indicate 1.5 × IQR. The number of independent samples is indicated at the top. **d** and **e,** Methylation levels (**d**) and segmented copy number values of all CpGs investigated using locally estimated scatterplot smoothing (LOESS) in groups of LGMT DICER1, SARC DICER1 and PIS DICER1 and (**e**) of all CpGs investigated in DICER1 associated mesenchymal tumors (LGMT DICER1, SARC DICER1 and PIS DICER1) according to groups of segmented copy number values. (**d**) Dashed lines mark copy number values of -0.1 and 0.1. The 95% confidence interval is shaded in grey. (**e**) Black lines mark the median, edges of boxes denote IQR, and vertical lines indicate 1.5 × IQR. The number of independent samples is indicated at the top. **f** and **g**, Difference (Δ) in methylation according to genome-wide distribution of differentially methylated probes (DMPs) identified in groupwise comparisons between (**f**) SARC DICER1 and LGMT DICER1, and between (**g**) PIS DICER1 and SARC DICER1. Dashed lines mark the mean difference in methylation according to genomewide distribution of DMPs. Fig. S5f and g are corresponding to Fig. 4d. **h** and **i**, Visualization of functional enrichment (gene ontology) analysis of genes overlapping with differentially methylated regions identified in groupwise comparisons between (**h**) LGMT DICER1 vs. SARC DICER1, as well as (**i**) SARC DICER1 vs. PIS DICER1. Supplementary Fig. 5h and i are corresponding to Fig. 4f. BP = biological process, MF = molecular function, CC = cellular component, FDR = false discovery rate.

Diagnoses	Abbreviation	n	*DICER1* status known		*DICER1* status unknown
**Total**		534	431		103
			***DICER1*-mut**	***DICER1*-wt**	
			176	255	
Alveolar rhabdomyosarcoma	ARMS	36	-	-	36
Anaplastic sarcoma of the kidney	ASK	4	4	-	-
Ciliar body medulloepithelioma	MEPL	12	4	3	5
Clear cell renal cell carcinoma	RCC	40	0	40	-
Cystic nephroma	CN	7	6	1	-
Embryonal rhabdomyosarcoma	ERMS	50	25	-	25
Embryonal tumor with multilayered rosettes	ETMR	26	6	20	-
High-grade endometrial stromal sarcoma	HGESS	9	-	-	9
Low-grade endometrial stromal sarcoma	LGESS	16	-	-	16
Lung adenocarcinoma	LAC	40	0	40	-
Müllerian adenosarcoma	MAS	27	6	21	-
Müllerian adenosarcoma/embryonal rhabdomyosarcoma	MAS/ERMS	9	4	5	-
Multinodular goiter	MG	14	14	-	-
Nasal chondromesenchymal hamartoma	NCMH	2	2	-	-
Papillary thyroid carcinoma	PCA	43	3	40	-
Pineoblastoma	PINB	41	11	30	-
Pleuropulmonary blastoma	PPB	21	21	-	-
Primary intracranial sarcoma DICER1	PIS DICER1	26	26	-	-
Pulmonary blastoma	PB	2	2	-	-
Sertoli leydig-cell tumor	SLCT	63	39	23	1
Spindle cell/sclerosing rhabdomyosarcoma	SRMS	11	-	-	11
Wilms tumor	WILMS	35	3	32	-

Supplementary Table 1: Institutional diagnoses and *DICER1* status of the study cohort (n = 534).

		PIS DICER1	NCMH	PB	PPB I/Ir	PPB II	PPB III	CN	ASK	ERMS
**Total**		26	2	2	6	6	9	7	4	24
**Location**	CNS	26	0	0	0	0	0	0	0	0
	Head and Neck	0	2	0	0	0	0	0	0	1
	Lungs	0	0	2	6	6	9	0	0	0
	Kidney	0	0	0	0	0	0	7	4	0
	Retroperitoneal	0	0	0	0	0	0	0	0	1
	Fallopian tube	0	0	0	0	0	0	0	0	1
	Uterus	0	0	0	0	0	0	0	0	21
**Age**	Range (years)	2 to 61	8	28 to 29	0.1 to 11	2 to 4	0.8 to 4	0.4 to 12	1.5 to 13	6 to 69
	Median (years)	6,2	NA	28,5	0,1	3	3	1,2	8,8	33,2
**Gender**	Female	14	1	2	1	2	4	4	2	23
	Male	12	1	0	5	4	5	3	2	1
	Ratio (M:F)	0,9	1	NA	5	2	1,25	1,3	1	0,04

Supplementary Table 2: Clinical characteristics of LGMT DICER1, SARC DICER1 and PIS DICER1 according to their institutional diagnoses (n = 86).

Methylation class		LGMT DICER1		SARC DICER1		PIS DICER1	
Number of patients	Total: 86	20		38		28	
Cases with available H&E	Total: 79 (92%)	18 (90%)		36 (95%)		25 (89%)	
Tumor configuration	Cystic	13	72%	1	3%	0	0%
	Solid, botryoid	5	28%	25	69%	0	0%
	Solid	0	0%	10	27%	25	100%
Cambium-like layer	Present	13	72%	25	69%	0	0%
Mesenchymal component	Fibrous stroma	5	28%	0	0%	0	0%
	Diffuse	13	72%	31	84%	8	32%
	Diffuse/fascicular	0	0%	5	14%	17	68%
Cellularity	Low	16	89%	2	5%	0	0%
	Intermediate	1	6%	22	61%	0	0%
	High	1	6%	12	32%	25	100%
Rhabdomyoblast-like features	Focal	1	6%	17	47%	12	48%
	Prominent	0	0%	10	27%	1	4%
Chondroid matrix	Focal	4	22%	9	24%	1	4%
	Prominent	1	6%	2	5%	1	4%
Anaplasia	Focal	1	6%	7	19%	11	44%
	Prominent	0	0%	6	16%	5	20%
Necrosis	Present	1	6%	15	41%	13	52%

Supplementary Table 3: Histopathological characteristics of mesenchymal tumors with DICER1 alteration according to LGMT DICER1, SARC DICER1 and PIS DICER1 cluster assignment (n = 79).

Methylation class	n	Mean_Global_No_CGI	Mean_Global_No_CGI_SD	Median_Global_No_CGI	Median_Global_No_CGI_MAD	Mean_CGI	Mean_CGI_SD	Median_CGI	Median_CGI_MAD	Mean_Promoter_region	Mean_Promoter_region_SD	Median_Promoter_region	Median_Promoter_region_MAD	Mean_Gene_body	Mean_Genebodies_SD	Median_Gene_body	Median_Genebodies_MAD
ERMS	6	0,596	0,031	0,592	0,04	0,217	0,009	0,221	0,007	0,312	0,018	0,313	0,027	0,601	0,029	0,597	0,035
HGESS	9	0,62	0,038	0,607	0,011	0,233	0,019	0,231	0,014	0,33	0,027	0,323	0,026	0,624	0,028	0,614	0,009
SARC DICER1	38	0,548	0,036	0,551	0,036	0,179	0,012	0,18	0,011	0,273	0,019	0,278	0,02	0,552	0,033	0,553	0,034
LGMT DICER1	20	0,606	0,03	0,61	0,027	0,181	0,016	0,18	0,009	0,301	0,012	0,302	0,011	0,601	0,026	0,605	0,023
MG	10	0,646	0,021	0,649	0,024	0,192	0,007	0,191	0,004	0,322	0,008	0,321	0,013	0,637	0,019	0,639	0,019
PIS DICER1	28	0,5	0,06	0,498	0,033	0,163	0,021	0,159	0,021	0,241	0,03	0,235	0,026	0,512	0,054	0,518	0,026
SLCT A	10	0,548	0,025	0,539	0,011	0,163	0,007	0,164	0,002	0,259	0,012	0,255	0,012	0,554	0,02	0,548	0,015

Supplementary Table 4: DNA methylation levels of global and CpG-dense regions for samples of LGMT DICER1, SARC DICER1, PIS DICER1, MG and SLCT DICER1, as well as ERMS and HGESS (related to Fig. 4a and Supplementary Fig. 5a - c).

Class vs.	Class	DMPs (total)	(0.1,1)	(-0.1,0.1)	(-1,-0.1)	DMRs (total)	(0.1,1)	(-0.1,0.1)	(-1,-0.1)
PIS DICER1	LGMT DICER1	360534	23414	90330	246790	1277	58	191	1028
SARC DICER1	LGMT DICER1	272772	29539	77525	165708	1011	89	212	710
PIS DICER1	SARC DICER1	257577	17255	110487	129835	629	40	221	368

Supplementary Table 5: Absolute numbers, and difference (Δ) in methylation of differentially methylated probes (DMPs) and regions (DMRs) identified in a group-wise comparison of LGMT DICER1, SARC DICER1 and PIS DICER1 (related to Fig. 4c and e).

Group (copy number value)	Mean	Mean_SD	Median	Median_MAD
< -0.1	0,438	0,151	0,43	0,163
-0.1 to 0.1	0,422	0,158	0,405	0,173
> 0.1	0,404	0,164	0,383	0,191

Supplementary Table 6: DNA methylation levels of all CpGs in segmented regions investigated in DICER1 associated mesenchymal tumors (LGMT DICER1, SARC DICER1 and PIS DICER1) according to groups of copy number status (related to Supplementary Fig. 5e).
