# Supplementary material for: Genomic characterization of DICER1-associated neoplasms uncovers molecular classes
Source: Nat Commun. 2023 Mar 25;14:1677. doi: 10.1038/s41467-023-37092-w (PMC10039902; doi:10.1038/s41467-023-37092-w)
Supplement: Supplementary file 3 — Reporting Summary [file 41467_2023_37092_MOESM3_ESM.pdf]

nature portfolio

Corresponding author(s): William D. Foulkes

Last updated by author(s): Feb 3, 2023

Reporting Summary

Nature Portfolio wishes to improve the reproducibility of the work that we publish. This form provides structure for consistency and transparency in reporting. For further information on Nature Portfolio policies, see our Editorial Policies and the Editorial Policy Checklist.

Statistics

For all statistical analyses, confirm that the following items are present in the figure legend, table legend, main text, or Methods section.

n/a	Confirmed	
	x	The exact sample size (*n*) for each experimental group/condition, given as a discrete number and unit of measurement
	x	A statement on whether measurements were taken from distinct samples or whether the same sample was measured repeatedly
	x	The statistical test(s) used AND whether they are one- or two-sided<br>*Only common tests should be described solely by name; describe more complex techniques in the Methods section.*
	x	A description of all covariates tested
	x	A description of any assumptions or corrections, such as tests of normality and adjustment for multiple comparisons
	x	A full description of the statistical parameters including central tendency (e.g. means) or other basic estimates (e.g. regression coefficient) AND variation (e.g. standard deviation) or associated estimates of uncertainty (e.g. confidence intervals)
	x	For null hypothesis testing, the test statistic (e.g. *F*, *t*, *r*) with confidence intervals, effect sizes, degrees of freedom and *P* value noted<br>*Give P values as exact values whenever suitable.*
x		For Bayesian analysis, information on the choice of priors and Markov chain Monte Carlo settings
x		For hierarchical and complex designs, identification of the appropriate level for tests and full reporting of outcomes
x		Estimates of effect sizes (e.g. Cohen's *d*, Pearson's *r*), indicating how they were calculated

Our web collection on statistics for biologists contains articles on many of the points above.

Software and code

Policy information about availability of computer code

Data collection	No Software was used to collect the data.
Data analysis	R version 4.1.2 (R Development Core Team, 2019): Champ version 2.24.0; ComplexHeatmap version 2.10.0; conumee version 1.12.0; copynumber version 1.24.0; ggplot2 version 3.3.6; limma version 3.34.5; minfi Bioconductor version 1.24.0; rstatix version 0.7.0; Rtsne version 0.13; survival version 3.3.-1; survminer version 0.4.9;<br><br>ANNOVAR software version 1.2.0<br><br>Integrative Genomic Viewer (IGV_2.7.2version)<br><br>The code to reproduce the main methylation analyses of this study has been deposited on figshare (https://doi.org/10.6084/m9.figshare.21739316.v1.).

For manuscripts utilizing custom algorithms or software that are central to the research but not yet described in published literature, software must be made available to editors and reviewers. We strongly encourage code deposition in a community repository (e.g. GitHub). See the Nature Portfolio guidelines for submitting code & software for further information.

Data

Policy information about availability of data

All manuscripts must include a data availability statement. This statement should provide the following information, where applicable:

- Accession codes, unique identifiers, or web links for publicly available datasets
- A description of any restrictions on data availability
- For clinical datasets or third party data, please ensure that the statement adheres to our policy

The methylation data generated in this study have been deposited in the NCBI GEO database under accession code GSE214568 [https://www.ncbi.nlm.nih.gov/geo/query/acc.cgi?acc=GSE214568]. The processed targeted DNA sequencing data are provided in the Supplementary data. However, participants did not agree to share the raw targeted DNA sequencing data. All other data is available in the Supplementary data. Source data are provided as a Source Data file.

Human research participants

Policy information about studies involving human research participants and Sex and Gender in Research.

Reporting on sex and gender	We performed a retrospective study with samples collected from multiple institutions. Sex and gender of patients was reported as indicated in clinical reports. Our study reports on the sex/gender distribution of patients affected by one of the three novel types of mesenchymal tumors with DICER1 alterations.
Population characteristics	The cohort analysed in this study is a compilation of individual tumor samples. Patients age ranged from 1 month to 69 years (median age 6.4 years).
Recruitment	Patients were not directly involved or recruited for this study. Tumor samples were collected from international peadiatric tumor and sarcoma reference centres.
Ethics oversight	This study was approved of by the Heidelberg Medical Faculty Ethics Commitee and was performed in accordance with the declaration of Helsinki. Samples were collected at McGill University-affiliated hospitals in Montreal, Quebec, Canada and the Institute of Pathology, University Hospital Heidelberg, mainly from collaborating institutions in accordance with local ethics review board regulations.

Note that full information on the approval of the study protocol must also be provided in the manuscript.

Field-specific reporting

Please select the one below that is the best fit for your research. If you are not sure, read the appropriate sections before making your selection.

☒ Life sciences ☐ Behavioural & social sciences ☐ Ecological, evolutionary & environmental sciences

For a reference copy of the document with all sections, see nature.com/documents/nr-reporting-summary-flat.pdf

Life sciences study design

All studies must disclose on these points even when the disclosure is negative.

Sample size	No sample-size calculation was performed. Samples were retrospectively collected, depending on their availability.
Data exclusions	No data was excluded from the analyses.
Replication	Genomic sequencing data were not reproduced due to limited tumor material available.
Randomization	The experiments were not randomized.
Blinding	Investigators were blinded to sample allocation during histopathological evaluation. Investigators were not blinded to sample allocation during all other computational experiments.

Reporting for specific materials, systems and methods

We require information from authors about some types of materials, experimental systems and methods used in many studies. Here, indicate whether each material, system or method listed is relevant to your study. If you are not sure if a list item applies to your research, read the appropriate section before selecting a response.

Materials & experimental systems

n/a	Involved in the study
☒	☐ Antibodies
☒	☐ Eukaryotic cell lines
☒	☐ Palaeontology and archaeology
☒	☐ Animals and other organisms
☒	☐ Clinical data
☒	☐ Dual use research of concern

Methods

n/a	Involved in the study
☒	☐ ChIP-seq
☒	☐ Flow cytometry
☒	☐ MRI-based neuroimaging
