# Supplementary material for: Genomic characterization of DICER1-associated neoplasms uncovers molecular classes
Source: Nat Commun. 2023 Mar 25;14:1677. doi: 10.1038/s41467-023-37092-w (PMC10039902; doi:10.1038/s41467-023-37092-w)
Supplement: Supplementary file 4 — Description of Additional Supplementary Files [file 41467_2023_37092_MOESM4_ESM.pdf]

Description of Additional Supplementary Files

Supplementary Data 1
Description: Case-by-case information on availability of DNA methylation data, methylation cluster assignment and DICER1 mutational status for the study cohort (n = 534).

Supplementary Data 2
Description: Case-by-case clinical and molecular characteristics of LGMT DICER1, SARC DICER1 and PIS DICER1 (n = 86).

Supplementary Data 3
Description: List of genes that were included in targeted sequencing.

Supplementary Data 4
Description: Variants called by targeted DNA sequencing of LGMT DICER1, SARC DICER1 and PIS DICER1 (n = 80).
